# Participatory organizational intervention for improved use of assistive devices for patient transfer: study protocol for a single-blinded cluster randomized controlled trial

**DOI:** 10.1186/s12891-016-1339-6

**Published:** 2016-12-20

**Authors:** Markus D. Jakobsen, Birgit Aust, Johnny Dyreborg, Pete Kines, Maja B. Illum, Lars L. Andersen

**Affiliations:** 1 National Research Centre for the Working Environment, Lersø Parkalle 105, DK-2100 Copenhagen, Denmark; 2 Department of Health Science and Technology, Physical Activity and Human Performance group, SMI, Aalborg University, DK-9220 Aalborg, Denmark; 3 Region Midtjylland, Koncern HR Fysisk Arbejdsmiljø, DK-8800 Viborg, Denmark

**Keywords:** Musculoskeletal disorders, Objective measure, Patient lift, Occupational injury, Accidents at work, Process evaluation, Occupational health, Health care, Back pain

## Abstract

**Background:**

Epidemiological studies have shown that patient transfer is a risk factor for back pain, back injuries and long term sickness absence, whereas consistent use of assistive devices during patient transfer seems to be protective. While classical ergonomic interventions based on education and training in lifting and transferring techniques have not proven to be effective in preventing back pain, participatory ergonomics, that is meant to engage and motivate the involved parties while at the same time making the intervention maximally relevant, may represent a better solution. However, these findings are largely based on uncontrolled studies and thus lack to be confirmed by studies with better study designs. In this article, we present the design of a study which aims to evaluate the effect and process of a participatory organizational intervention for improved use of assistive devices.

**Methods:**

The study was performed as a cluster randomized controlled trial. We recruited 27 departments (clusters) from five hospitals in Denmark to participate in the study. Prior to randomization, interviews, observations and questionnaire answers (baseline questionnaire) were collected to gain knowledge of barriers and potential solutions for better use of assistive devices. In April 2016, the 27 departments were randomly allocated using a random numbers table to a participatory intervention (14 clusters, 324 healthcare workers) or a control group (13 clusters, 318 healthcare workers). The participatory intervention will consist of workshops with leaders and selected healthcare workers of each department. Workshop participants will be asked to discuss the identified barriers, develop solutions for increasing the use of assistive devices and implement them in their department. Use of assistive devices (using digital counters -, primary outcome, and accelerometers and questionnaire - secondary outcome), perceived physical exertion during patient transfer, pain intensity in the lower back, occurrence of work-related back injuries during patient transfer, organizational readiness to change, knowledge on how to perform proper patient transfer, social capital and work ability (secondary outcomes) were assessed at baseline and will also be assessed at 1 year follow-up. Process evaluation will be based on qualitative and quantitative data to assess the implementation, the change process, and the impact of context aspects.

**Discussion:**

The study will evaluate the effect and process of a participatory intervention on improving the use of assistive devices for patient transfer among hospital healthcare workers. By using cluster-randomization, as well as process- and effect evaluation based on objective measures we will contribute to the evidence base of a promising intervention approach.

**Trial registration:**

ClinicalTrials.gov (NCT02708550). March, 2016.

## Background

Musculoskeletal pain and musculoskeletal injuries, particularly in the lower back, represent a major problem for healthcare workers and their leaders in terms of increased individual suffering and long term sickness absence [1–3].

In a study including 8000 Danish eldercare workers, 23% reported having at least 30 days of pain during the last year in the lower back [4]. Even higher numbers have been reported in a recent review of the prevalence of musculoskeletal disorders showing that 44% of American healthcare workers working in hospital settings are suffering from chronic musculoskeletal pain (at least 3–6 months) in the lower back. The review also concluded that the average injury rate across studies was 14% for reported lower back injuries, and 20% for injuries with lost work days [3]. In support of this, Koppelaar and colleagues observed that 58% and 65% of nurses working in Dutch nursing homes and hospitals, respectively, had experienced musculoskeletal complaints in the past 12 months [5]. This suggests that hospital nurses may be at an even higher risk of developing musculoskeletal pain and injuries compared with nurses working in nursing homes.

A recent systematic review stated that patient handling/transfer represents the highest risk factor for developing lower back disorders (injuries and lower back pain) among nurses and nursing assistants [6]. The relationship between patient transfers and back disorders is supported by biomechanical investigations demonstrating that patient transfers often involve loadings on the spine that exceeds the 3400 N safety limit recommended by the National Institute for Occupational Safety and Health [7–10]. Further, support is given by studies demonstrating that patient transfer is the precipitator of 72–89% of all filed musculoskeletal injuries among healthcare workers in hospital settings [2, 11, 12]. Moreover, a study from our research group found that performing patient transfer on a daily basis is prospectively associated with almost a doubling of the risk of sustaining a back injury [13].

A traditional strategy to reduce pain and work-related injuries among healthcare workers is education and training in lifting and transferring techniques [14, 15]. Although this approach is vastly used, a recent systematic review on educational interventions to prevent low back pain in a variety of different job groups, including healthcare workers, concluded that training employees in proper material handling techniques or providing them with assistive devices are not effective interventions by themselves in preventing back pain [16]. The absence of effects may be due to too simplistic models of health behavior change, assuming that the information provided in the intervention by itself leads to a change in knowledge, attitudes or skills. In fact, more comprehensive approaches for reducing the high risk of work-related musculoskeletal injuries among healthcare workers developed in recent years are promising. For example, an Australian study found a 24% reduction in reported back injury claims after imposing a ’no lifting policy’, where nurses across an entire healthcare system were instructed to use assistive devices for every patient transfer [17]. In addition to eliminating or minimizing manual handling when transferring patients, the provision of patient handling equipment and education in ’no lifting techniques’, the program also included a variety of other activities for example strategies to support cultural change and ownership by nurses, as well as organizational commitment. Importantly, consequent use of assistive devices was in a prospective cohort study associated with reduced risk for sustaining back injuries among healthcare workers from Denmark [13].

Another promising approach to reducing work related pain and injuries is the use of employee involvement through participatory ergonomic (PE) interventions [18, 19]. PE interventions have been defined as *“the involvement of people in planning and controlling a significant amount of their own work activities, with sufficient knowledge and power to influence both processes and outcomes in order to achieve desirable goals”* [20]. In another study, Kourinka defined PE as *“practical ergonomics with participation of the necessary actors in problem solving”* [21]. Thus, the PE approach is meant to engage and motivate the involved parties while at the same time making the intervention maximally relevant. In a more recent study, Garg and Kapellusch found a 60% reduction in patient handling injuries after increasing the number of assistive devices and implementing a participatory ergonomics intervention specifically aiming at reducing patient handling injuries [15]. The PE intervention used in the study by Garg and Kapellusch was based on a framework by Haines et al. consisting of nine dimensions, including for example permanence of initiatives, involvement, level of influence and decision-making power [22]. Although, the PE approach seems promising, most studies testing this approach lack proper randomization or a control group [18, 19]. Hence, there is a need to investigate PE approaches in high-quality randomized controlled trials.

Great efforts have been made to increase knowledge, availability and use of assistive devices to reduce work-related physical strain due to patient transfers in hospitals in Western countries [23–25]. Nevertheless, we recently found in a pilot survey among more than 300 Danish nurses and nurses’ aides, showing, that only a third of the employees often used assistive devices when deemed necessary during patient transfer. However, because respondents to questionnaires may be influenced by recall bias these data should be interpreted with caution. Accordingly, real-time measures using i.e. observations or objective measures of the use (movement) of the assistive device, using e.g. sensors, can provide a more valid measure. By using real-time observations Koppelaar et al. reported a somewhat more positive prevalence among Dutch nurses, where 68% of the nurses working in nursing homes and 59% working in hospitals used ergonomic devices when deemed necessary [5]. Still, it seems that potential barriers for using assistive devices may be even more apparent among healthcare workers in Danish hospitals. Studies have shown that these barriers include: the time required to use the assistive devices; convenience and easy accessibility; proper guidelines, management cooperation and support; cost; lack of proper training; motivation; and reluctance to use the devices for patient transfer hereby including concerns about patient comfort, safety, rehabilitation and integrity [5, 26–30]. As hospitals in Denmark and many other western countries, seem to be equipped with the necessary assistive devices, a lot of the barriers may therefore either be related to management support, guidelines or individual motivation for using assistive devices. However, the barriers may be different from department to department, making the PE approach highly relevant to customize the intervention. As PE interventions have shown to increase ownership and understanding of these challenges [18], although from non-randomized trials, implementing PE interventions in hospitals represent a promising strategy to develop solutions for increasing the use of assistive devices.

The purpose of this study is to evaluate a participatory organizational intervention for improved use of assistive devices in a cluster randomized controlled trial. The primary outcome is the change in use of assistive devices at department (cluster) level.

## Methods

### Study design and randomization

A two-armed parallel-group, single-blind, cluster randomized controlled trial with allocation concealment is conducted among healthcare workers from five Danish hospitals situated in Zealand (*n* = 4) and Jutland (*n* = 1 large hospital situated at 4 different geographical locations). Clusters are hospital departments and hospital units that work together as separate entities. Thus, cluster randomization was chosen to avoid contamination between individuals of each group. The randomization was performed using a random numbers table by a person without knowledge of the status of each department. For practical purposes, each department was assigned a random number, the columns were sorted ascendingly, and every other department in that order was assigned to intervention and control, respectively. Immediately after randomization we informed, by email, the participants at the respective departments and their manager about group allocation. The departments were parallel assigned to a 1 year participatory intervention to increase the use of assistive devices, or to a control period of 1 year. The intervention duration is from April 2016 to April 2017. The reporting of the study will follow the CONSORT statement for cluster trials [31] and SPIRIT [32, 33] statements.

### Participants

In April 2016, 27 departments (clusters) with 642 healthcare workers located at five different Danish community hospitals were allocated to a participatory intervention or a control group. The inclusion criteria were departments with healthcare workers working with daily patient transfer. Baseline characteristics of employees belonging to the 14 departments in the intervention group and to the 13 departments in the control group are presented in Table 1.

**Table 1:** Characteristics of the two groups. Values are presented as numbers, percentage and mean (SD) from the participants who answered the baseline questionnaire

	Intervention	Control
Departments (N)	14	13
Employees (N)	324	318
Women (N)	289	285
Men (%)	12	12
Age (years)	43 (12)^a^	40 (12)
Height (cm)	169 (7)	170 (7)
Weight (kg)	70 (15)	71 (14)
BMI (kg∙m^-2^)	24 (4.6)	25 (4.4)

^a^ difference between groups at baseline, *P* < 0.05

### Recruitment

**Fig. 1:**
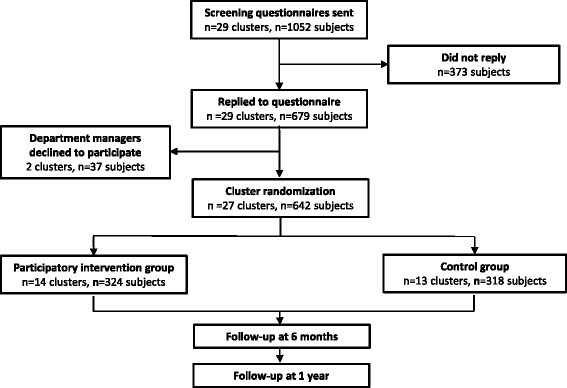
Flow-chart of the participants who received the baseline questionnaire

The initial recruitment of hospitals took place when the grant application was written in 2014. Final recruitment of hospitals and departments took place throughout 2015. Initial contact was taken to the work environment and health and safety staff from eleven hospitals, who were asked if the project could be of interest for departments at their hospital. Four hospitals were working with other projects at the moment, and therefore were not interested in participating. The hospital’s work environment staff from the five remaining hospitals who were interested was asked to point out the hospital departments that performed daily patient handling using assistive devices. A total of 35 departments were pointed out from the five hospitals, of which 29 initially were interested in participating. In February 2016 a baseline questionnaire was e-mailed to the 1052 healthcare workers (nurses and nursing aids) employed in the 29 department. In total 679 healthcare workers (65%) from the 29 departments replied to the questionnaire. After the questionnaire survey two departments decided not to participate due limited time for participation in the study. The study population therefore consists of 27 departments with 642 participants who answered the baseline questionnaire. The flow of clusters and participants is illustrated in Fig. 1.

### Phase 1: assessment of barriers and potential solutions

**Fig. 2:**
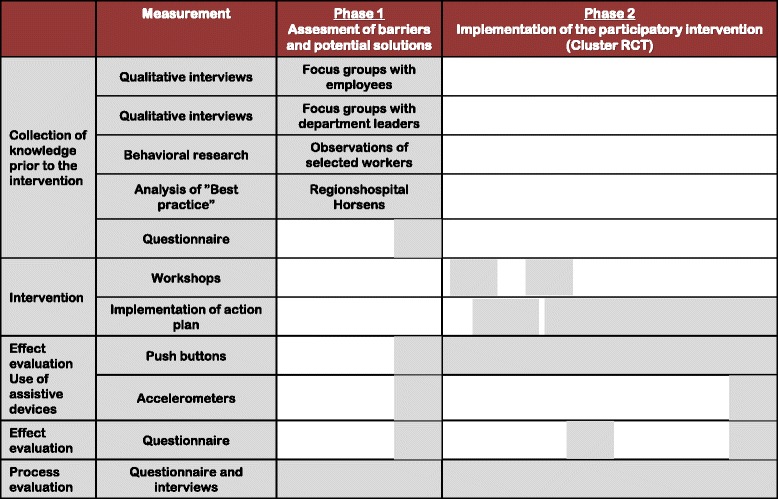
Elements, measurements and phases of the study

From September 2015 to February 2016 and before randomization a range of different measures were used to examine the motives and barriers for the use of assistive devices for patient transfers and to collect ideas for potential solutions that all could be used in the subsequent participatory intervention. The measures included a questionnaire survey in all departments participating in the study, interviews with department leaders and employees, observations of patient transfers in selected departments as well as the analysis of the experiences of a “best practice” hospital. Details about these measures are described in the following paragraphs. The different phases of the intervention are presented in Fig. 2.

#### Interviews

To get a more profound understanding of the potential barriers for the use of assistive devices semi-structured qualitative interviews were performed at each of the five hospitals with selected employees and department leaders. All 27 participating departments were invited to participate in the interviews. More specifically, an e-mail was sent to all department leaders asking if they were willing to be interviewed, and if they could find three to five employees in their department who also would be willing to be interviewed. Positively replying department leaders were contacted to find a date and time for the interviews that fit with the schedules of the interviewees. Department leaders and employees were interviewed separately. Department leaders were interviewed individually or together with another department leader from the same hospital. Employees from one or two departments from the same hospital were interviewed in groups.

Interview guides were developed for the two types of interviews (department leader(s), groups of employees). Both interview guides were based on knowledge identified in previous studies focusing on barriers for using assistive devices, and on the results of a pilot-survey investigating barriers for use of assistive devices in three Danish hospitals [5, 26–30]. All interviews started with a general introduction about the intervention study and the purpose with the interview, and ended with information about the next steps in the intervention study.

The interviews with the department leaders (eight department leaders from five hospitals) focused on aspects such as the hospitals, the department leaders’ and the employees’ general views regarding the use of assistive devices. It also included questions about experiences and challenges with the use of assistive devices, as well as questions about activities that support employees in the use of assistive devices. Other questions focused on the availability and possibilities for ordering additional devices. The final part of the interview addressed the department leaders’ role with regard to employees’ use of assistive devices, as well as suggestions for changes to improve the use of assistive devices.

The group-interviews with 23 employees (13 departments from five hospitals) focused on identifying barriers for use of assistive devices and development of suggestions. Out of 16 pre-defined barriers (example e.g. ‘time’ (to find and get assistive devices) or ‘space’ (not enough space in the patients rooms for using assistive devices)), employees were asked to identify the three barriers that they felt were most pronounced in their department, and to give examples of how these barriers kept employees from using assistive devices more frequently. Employees could also choose barriers that were not represented in the 16 pre-defined barriers. In the last part of the interview employees were asked about what they thought needed to be done in order to improve the use of assistive devices in their department.

#### Questionnaire

The baseline questionnaire was sent to all employees and leaders (who we received E-mail addresses on) of the participating departments in February 2016. To assess barriers and potential solutions, we included questions about the following topics in the baseline questionnaire: 1) Self-efficacy to increase the use of assistive devices, 2) motivation and barriers and potential solutions for increasing the use of assistive devices, and 3) knowledge of the availability of assistive devices at the department.

#### Observations

Observations of the healthcare workers’ behavior before and during a patient transfer were conducted in selected departments. The purpose of the observations was to identify barriers for using assistive devices that are difficult to identify in surveys or interviews. We asked 29 departments of which six departments from two hospitals were willing to participate in the observations. Four hours of observation were conducted three times at each of the six departments by students who had been trained in performing workplace observations. The observers were either alone or in teams of two. The observations were based on the ‘nudging’ philosophy to overcome motivation and ease in using assistive devises. Nudging is assumed to alter the choice architecture which will influence people’s behavior in a predictable way [34]. Nudges can be seen as ways of influencing healthcare workers’ choices, in this case the use of assistive devices, without necessarily limiting alternative decisions by social sanctions, as is the case with a no lift policy. Although barriers were identified, the observations did not lead to any specific ideas for nudges. Therefore the observations did not contribute to suggestions for changes to be considered by the workshop participants. Longer periods of observations may be needed to fully understand the barriers for using assistive devices that can be solved by developing nudges.

#### Analysis of “best practice” hospital

To obtain knowledge on potential solutions for increasing the use of assistive devices we performed an analysis of a "best practice" hospital. We chose this specific hospital because they, during the last 8 years, have had success in increasing the use of assistive devices and lowering the number of accidents through implementation of “Competent Mobilization” (CM), which included participatory interventions for increasing the use of assistive devices as well as financial aid for purchase of additional assistive devices.

To get an understanding of how this hospital succeeded in increasing the use of assistive devices, semi-structured qualitative interviews were performed with: 1) employees, ergo-coaches (employees who are able to educate others in the use of assistive devices during patient transfer) and department leaders (*n* = 5), 2) work environment coordinators/consultants (*n* = 2), and 3) hospital directors (*n* = 2). Each interview lasted approximately one hour. Department leaders and employees were interviewed in groups, whereas work-environment consultants and hospital directors were interviewed individually. The interview guide used for these interviews were inspired by the interview guides presented above, however with a specific focus on how the barriers for using assistive devices were overcome, and what had been done to achieve a lasting effect. Both types of interviews focused on the different aspects of the implementation of CM. The interview guides included questions on: a) how CM affected the general view on use of assistive devices, b) in regards to use of assistive devices, what are the main differences before and after CM, c) which activities took place in CM, d) how were the employees supported in their use of assistive devices, e) what is the current availability and possibilities for ordering additional devices, both practically and economically. The interviews with the hospital directors also addressed the directors’ role in assuring a high level in use of assistive devices among employees. The interviews with the department leaders, employees and ergo-coaches specifically focused on how they succeeded in overcoming the barriers for using assistive devices.

### Phase 2: participatory intervention

A characteristic feature of participatory ergonomic interventions is the formation of a problem solving team, which is given ownership and insight of the challenges at hand that need to be solved [19]. The current intervention will consist of two two-hour workshops where the problem solving team consisting of selected employees (nurses, nursing aides and porters), leaders and the hospital’s health and safety consultants will be asked to develop and implement an action plan consisting of multiple solutions to increase the use of assistive devices in their department (at cluster level) (Fig. 2 - Phase 2). The main aim of the first workshop is to kick-start the participatory process by: a) letting the participants brainstorm on barriers and potential solutions for improving the use of assistive devices, and b) subsequently develop a simple action plan for implementing one of these solutions. Approximately two weeks before workshop I, employees in intervention departments will receive a report via e-mail. The report summarizes selected results of the baseline questionnaire in order to provide employees and their department leaders with information about specific challenges with regard to the use of assistive devices in their department. Based on discussions of the report and presentation of barriers and potential solutions collected through the aforementioned interviews and analysis of “best practice”, participants will then be asked to identify and discuss potential solutions for increasing the use of assistive devices at the department. The workshop participants are then asked to select one achievable solution and implement it over the course of the following weeks. Approximately 3 months after the first workshop all departments will be invited to a second and final workshop. The aims of workshop II is twofold: a) to discuss the status and experiences with the implementation of the solution developed in workshop I, and b) develop an action plan for the implementation of 1–5 additional solutions. The decision about which suggestions to choose should be based on what workshop participants are most motivated for, and which are considered to have the largest effect on improving the use of assistive devices in the 6–9 months after workshop II.

At both workshops participants will be asked to set deadlines for the implementation of their suggestions, and to appoint persons responsible for the implementation of each of the chosen solutions. The workshop participants are also asked to present and discuss the action plan, and the reasons for choosing the solutions at department meetings in the weeks following the workshops. The researchers will on a regular basis check (using small surveys and telephone and E-mail contact) if the department’s action plan deadlines are met or, if not, encourage them (E-mail or telephone coaching) for meeting these deadlines.

All workshop participants will be informed about the purpose and content of the project and will be asked, by the researchers in charge of the workshops, to give their written informed consent to participate in the study. The participation in the workshops is voluntary and the participants are free to withdraw from the study at any time.

### Control group

The 13 departments randomized to the control group will not receive any intervention, and will be encouraged to continue their normal working procedures throughout the study period.

### Process evaluation

Through process evaluation we aim to: 1) document if the different parts of the interventions are actually implemented, 2) follow the change process, as well as 3) detect and understand eventually divergent activities and unexpected outcomes. The process evaluation framework used in this study is inspired by different process evaluation approaches [35–37], which partly overlap but also focus on specific aspects only represented in one or two of these approaches. The approach developed by Saunders et al. (2005) focusses primarily on aspects that help to document the degree of implementation, i.e. to what degree the intervention was implemented as planned. It consists of the following aspects: fidelity (extent to which the intervention was implemented according to the principles of the intervention), dose delivered (amount or number of intended units of each intervention or component delivered or provided by interventionists), dose received/exposure (extent to which participants actively engage with, interact with, are receptive to, and/or use materials or recommended resources), dose received/satisfaction (participants satisfaction with program, interactions with staff and/or investigators), reach (participation rates of the different activities), recruitment procedures used to approach and attract participants at individual or organizational levels, context (aspects of the environment that may influence intervention implementation or study outcomes).

The model of Nielsen and Randall (2013) overlaps partly with the aspects from the Saunders approach, but also adds important components, for example the aspect of “mental models”, i.e. the participants’ attitudes to the intervention before and under the intervention (e.g. readiness for change) which we consider particularly important for the understanding of participatory interventions [38]. In addition, the framework developed by Fridrich et al. (2015) overlaps with the other two approaches, but adds a more thorough evaluation of context that goes beyond a conceptualization of context as “a static, non-changeable boundary condition” (page 4) [37]. Instead they recommend distinguishing between ‘omnibus context’, describing the overall setting in which the intervention takes place and which typically are difficult to change, and the ‘discrete context’ which refers to specific individual, leader, group and organizational aspects directly relevant to the implementation and change process, and which in principle can be changed and should be a target for change.

Based on these three approaches we will focus on answering the following questions:

- To what degree was the intervention implemented as planned? (e.g. were two workshops per department conducted? Were action plans developed?)
- How did the workshop-participants and all employees at the participating departments perceive the intervention? (Here we will particularly focus on if employees felt that improvement suggestions were meaningful, and if they were involved in the implementation of the improvement suggestions.)
- Did the mental models of the participants have an influence on the implementation process (e.g. employees’ expectation that use of assistive devices can be increased in the department)?
- Did context aspects (omnibus and discrete context) have an influence on the implementation, and if yes, in which way and how it was handled?

Our data sources consist of qualitative and quantitative measures including three questionnaire surveys (before (baseline), during (follow-up I) and after the intervention (follow-up II)), detailed notes from the two workshops in each department, project documentation about activities and materials delivered to the intervention departments and implementation status documentation (once a month after workshop 2). While all of these measures will be conducted in all intervention departments, a more detailed qualitative assessment is only possible in selected departments due to budget restraints. Based mainly on information from the monthly implementation status documentation, two departments with a high level, and two departments with a low level of implementation will be selected for focus group interviews with employees, which will be conducted after the end of the intervention period. In these departments employees will be asked to elaborate on the implementation process and why implementation succeeded or did not succeed.

The methods for the process evaluation will be developed continuously during the project period to enable us to focus on unforeseen implementation challenges or aspects we wish to assess further. Thus, the final process evaluation may divert somewhat from the present evaluation plan (e.g. total number of interviews) or methods used.

### Blinding

Due to the interventional trial design, participants and researchers managing the workshops cannot be blinded to group allocation. However, outcome assessors and quantitative data analysts will be blinded to group allocation.

### Outcome measures

Outcomes are objectively measured by digital push-button counters and by accelerometers at baseline and 1 year follow-up, and by questionnaire survey at baseline, after 6 months and at 1 year follow-up.

#### Primary outcome measure

The primary outcome is the use (at department level) of assistive devices during the entire 1 year follow-up adjusted for the use at baseline (4 weeks prior to the intervention). The use (frequency and total amount) of assistive devices (i.e. lifts, sliding sheets or patient transporters) is measured continuously during the entire project-period with digital push-button counters. Two digital push-button counters will be placed next to the doorframe of the exit door in each patient room. The healthcare workers are encouraged to push one of the following two buttons every time they leave the room after having performed patient transfer; button 1 (green) which is labelled with *“Press this button if you used the necessary assistive devices for your patient transfer”* and button 2 (red) which is labelled with *“Press this button if you did not use the necessary assistive devices for your patient transfer”*. The ratio between the number of Button 1 counts and the total number of counts (Button 1 + 2) will be calculated for each set of counters and used as the primary outcome. To increase the employees’ motivation for pressing the buttons, frequent motivational emails to all participants, telephone calls to the departments and occasional personal attendance will be administered throughout the study period.

#### Secondary outcome measures

Secondary outcome measures are: 1) objectively measured use of assistive devices using accelerometers (at the department level), and by questionnaire (at the individual level accounting for cluster), the change from baseline to 1 year follow-up in 2) musculoskeletal pain intensity during the last week (back, neck and shoulder), 3) perceived physical exertion during work, 4) occurrence of work-related back injuries during patient transfer, 5) work ability, 6) social capital, 7) knowledge on how to perform proper patient transfer, and 8) organizational readiness to change. To measure short-term effects the questionnaire will also be distributed after 6 months. During each questionnaire round, participants with non-response will receive up to two reminders to reply.

Besides using pushbuttons for measuring the use of assistive devices we will also measure the use (frequency and total amount) of assistive devices (i.e. lifts or patient transporters) with accelerometers (Actigraph, Florida, US), normalized to the number of employees with patient transfer in each department. The accelerometers will measure the movement of the assistive device, and will be discretely placed on patient transporters/lifts for periods of three weeks at baseline, and at 1 year follow-up. The use of sliding pieces and other disposable assistive devices will not be measured using accelerometers in this study. Using questionnaires, the use of assistive devices will, furthermore, be obtained by asking: *“Do you transfer patients without the necessary assistive devices? (Think of situations where you should have used assistive devices)”*. Subjects will reply on a 5-point scale: 1) “0 out of 4 transfers (i.e. almost never)”, 2) “1 out of 4 transfers”, 3) “2 out of 4 transfers”, 4) “3 out of 4 transfers” and 5) “4 out of 4 transfers (i.e. every time)”.

Pain intensity in the lower-back will be rated subjectively using a 0–10 modified visual analog scale, where 0 indicates “no pain at all” and 10 indicates “worst pain imaginable” [39, 40]. Drawings from the Nordic questionnaire for the analysis of musculoskeletal symptoms are used to define the body regions of interest [41].

Perceived physical exertion during work will be rated using a Borg’s Rate of Perceived Exertion (RPE) scale, at baseline, 6-months and after 1-year follow-up. The Borg RPE scale has been validated in many different contexts to measure actual exertion, e.g. perceived exertion during manual handling tasks [42, 43]. The participants will be asked the following question: *“In general, how would you rate your physical exertion while transferring the patients?”.* Subjects will reply on a scale with nine exertion levels taken from the BORG CR10 scale (0–10): “Nothing at all” (RPE = 0), “very, very light” (RPE = 0.5), “very light” (RPE = 1), “light” (RPE = 2), “moderately strenuous” (RPE = 3), “somewhat strenuous” (RPE = 4), “strenuous” (RPE = 5), “very strenuous” (RPE = 7), and “very, very strenuous” (RPE = 10) [42, 44].

The participants will also be asked the following question regarding occurrence of low-back injuries: *“Have you within the last 12 months injured your back during patient transfers? (Think of situation where the pain appeared suddenly and unexpectedly)”.*

We will, furthermore, measure work ability using three items of the seven item work ability index score (WAI): 1) current work ability compared with the lifetime best, 2) work ability in relation to the physical demands of the job, and 3) work ability in relation to the number of physical demands of the job [45, 46].

The participants will also reply to a questionnaire concerning bonding (within department) and linking (between leaders and employees) social capital [47]. The questionnaire has been developed and validated with the use of qualitative interviews at the National Research Centre for the Working Environment in Copenhagen, Denmark [48]. We have previously shown that implementing interventions on a department level (weekly exercises during working hours) can increase the bonding among healthcare workers [47].

Lastly, we will measure organizational readiness to change [49], as well the participant’s knowledge on how to perform proper patient transfer, using custom made questions developed for this project by the participating hospitals’ health and safety consultants and the Danish Working Environment Authority.

### Sample size

The sample size calculation was based on the primary outcome. A priori power analysis based on previous measurements revealed that 13 clusters in each group (26 departments in total) for 95% power, SD of 10% and a minimal relevant group-difference in the use of assistive devices of 15%, is sufficient to test the null-hypothesis of equality (α = 0.05).

### Statistical analysis

All statistical analyses will be performed using the SAS statistical software for Windows (SAS Institute, Cary, NC). For the primary outcome, use of assistive devices during the entire 1-year follow-up will be evaluated using linear mixed models. Analyses will be adjusted for use of assistive devices during baseline. Cluster is entered in the model as a random factor. We will perform all statistical analyses in accordance with the intention-to-treat principle using a linear mixed model, which inherently accounts for missing values. An alpha level of 0.05 will be accepted as significant. Outcomes will be reported as between-group least mean square differences and 95% confidence intervals from baseline to follow-up.

### Adverse events

Although this is an organizational intervention, adverse events in relation to participation in the project will be registered with an open-ended question at the questionnaire at 6 months and 1 year follow-up. The question will be *“Have you experienced any adverse events – physical, mental or other - in relation to your participation in the project”*.

## Discussion

Studies have shown that barriers for using assistive devices are often related to individual motivation, lack of proper meaningful guidelines and management support [5, 26–30]. One theory is that these barriers for using assistive devices among healthcare workers may be a result of reduced worker involvement [50]. As participatory interventions have shown to increase ownership and understanding of these challenges [18], we decided to design a study where healthcare workers and leaders, using a participatory approach, develop solutions for increasing the use of assistive devices in their department. Consequently, the present study will, in a cluster randomized controlled trial, provide documentation on: 1) the efficacy of an organizational participatory ergonomic intervention for, objectively measured, increased use of assistive devices, and 2) how to improve the use of assistive devices for patient transfer among hospital healthcare workers.

Although participatory approaches have been shown to reduce the number of work-related injuries, the majority of the studies are non-randomized or without a control group [18]. The lack of randomized controlled trials may be due to the relatively low incidence rate of work-related injuries during patient transferring, and thus the need for sufficient statistical power (the annual incidence rate of back injuries is less than 4% among Danish healthcare workers [13]). Accordingly, Burdorf et al. suggested that finding statistically significant results in injury rates in a randomized controlled trial on reduction of manual patient transfer would require a sample size of more than 10.000 healthcare workers [51]. Therefore, other solutions need to be found. As consistent use of assistive devices for patient transfer may reduce the risk of work-related injuries and musculoskeletal pain [13, 15, 17], the present study will use objective measures of the use of assistive devices for patient transfer as a proxy outcome for injury reduction/prevention that is more accessible for randomized controlled trials. Thus, we hypothesize that improving the use of assistive devices per patient transfers will reduce the total number of manual lifts/transfers, and thus the risk of patient transfer injuries.

### Strength and limitations

Compared to previous studies using self-reported data [15, 29, 50], a strength of this study is the objectively measured use of assistive devices. However, since it is not possible to measure the use of all devices using accelerometers (i.e. sliding sheets), there are still limitations to using this approach. For that reason, we chose to encourage the employees to press interactive digital push-button counters whenever they leave a patient-room after having performed a patient transfer. Using this method we hope to be able to assess the actual relationship between patient transfers with and without the use of any type of assistive device in situations where use of assistive devices was deemed necessary. However, as this approach is an objective measure that is based on individual subjective motivation for pushing the buttons, questionnaires regarding the healthcare workers’ use of the push-buttons, and observations of a working day and their use of the buttons after performing patient transfer will be conducted to validate this method. A limitation of this method is, however, that it’s not possible to distinguish between the types of assistive devices used and not used. To partly accommodate for this, we will also measure the use of assistive devices such as lifts and mobile patient transporters using accelerometers. However, a full analysis of the total use of all types of assistive devices is not possible using this setup.

Besides an effect evaluation of study outcomes, another strength of this study is the incorporation of a process evaluation assessing the implementation, the change process and the impact of context aspects. Through this comprehensive evaluation we expect to gain insight on how to improve the use of assistive devices through participatory worker involvement. A final strength of this study design is the use of cluster randomization, which prevents contamination between individuals of the intervention and the control group.

## Abbreviations

CM: Competent mobilization
PE: Participatory ergonomic
RPE: Rate of perceived exertion
WAI: Work ability index score
